# Supplementary material for: Tectonically-driven oxidant production in the hot biosphere
Source: Nat Commun. 2022 Aug 8;13:4529. doi: 10.1038/s41467-022-32129-y (PMC9360021; doi:10.1038/s41467-022-32129-y)
Supplement: Supplementary file 1 — Supplementary Information [file 41467_2022_32129_MOESM1_ESM.docx]

**Supplementary Information for**

**Tectonically-driven oxidant production in the hot biosphere**

This file includes:

Supplementary Discussion

Supplementary Figures

Supplementary Tables

# 1. Supplementary Discussion

**Oxygen mass balance calculations**

The μmol O_2_ in the starting vials was estimated by summing together a) μmol of O_2_ in the ball mill during grinding, b) the μmol of O_2_ in the 6 mL vial headspace, and c) the added 4 mL of water.

To calculate the O_2_ that could be added during grinding in the ball mill, we carried out triplicate blank runs using the same protocol as for rock samples with the omission of the rock and the grinding balls (the latter to avoid excessive wear on the agate ball mill). After placing in the Planetary Ball Mill for 30 min at 500 rpm, the ball mill was transferred to the glove bag (<0.1 ppm O_2_) and a 1/8 inch Swagelok fitting containing a rubber septa was attached to the one of the ball lid valves. A 10 mL gastight syringe and needle was then used to sample 3 mL of gas from the ball mill, after pumping the syringe 6× to ensure the outlet valve gas was in equilibrium with the ball mill interior. This 3 mL of gas was injected into a 3 mL pre-evacuated Exetainer with double-wadded cap. The Exetainers were then removed from the glove bag and O_2_ measured using a needle-type Presens O_2_ sensor freshly calibrated against a 0% (sodium sulfite in water) and air. For comparison, the O_2_ was also measured in three Exetainers to which 3 mL of 99.999% N_2_ were added within the glove bag (Supplementary Table 1).

**Supplementary Table 1**. O_2_ measurements of blank ball mills runs compared to starting N_2_

|  | % O_2_ in Exetainers + 3 mL sampled from ball mill blank runs | % O_2_ in Exetainers + 3 mL N₂ |
| --- | --- | --- |
|  | 0.30 | 0.27 |
|  | 0.24 | 0.34 |
|  | 0.35 | 0.32 |
| mean | 0.30 | 0.31 |
| stdev | 0.06 | 0.04 |

The small concentration of O_2_ within the N_2_ filled Exetainers (mean of 0.31%) is most likely due to residual air within the evacuated vials prior adding samples. The lack of any significant difference (± 1 × standard deviation) between starting N_2_ gas and the gas measured in ball mill after blank runs indicates no detectable air contamination during grinding. However, due to the variation in the N_2_-filled Exetainers, we cannot rule out a small amount of contamination; even a small percent contamination of O_2_ in the ball mill could have an appreciable result given the relatively large volume of the ball mill (49.3 mL when filled with grinding balls and 45 g rock, assuming a rock density of 3.0 g mL^-1^) relative to the incubation vials (4 mL headspace). We therefore conservatively use a value of 3 × standard deviations of the blanks (0.12 %) as a maximum estimate of O_2_ contamination value during the ball milling. To calculate the nmol g^-1^ O_2_ equivalent, we use the ideal gas law (n = PV/RT), P = pressure of O_2_ in atmospheres (0.0012), V = volume of vial headspace (0.048 L), R = Gas Constant (0.08314426 L⋅bar⋅K^−1^⋅mol^−1^), and T = temperature (293.15° K), and divide by 45 g to normalise to rock mass within the ball mill, to give 54.7 nmol O_2_ g^-1^.

To calculate the O_2_ added in to vials in the headspace of the vials, we again use the ideal gas law (n = PV/RT), using an upper estimate of O_2_ in the glove bag of 0.1% (see Methods), where V = volume of vial headspace (0.006 L), R = Gas Constant (0.08314426 L⋅bar⋅K^−1^⋅mol^−1^), and T = temperature (293.15° K), and divide by 2 g to normalise to rock mass to give 1.2 nmol O_2_ g^-1^. To calculate the O_2_ added in the water we use the maximum dissolved O_2_ measured in the experiments (0.268 mg L^-1^, equivalent to 8.38 μmol L^-1^), normalize to 4 mL, and divide by 2 g to give 16.8 nmol g^-1^. We then add the O_2_ from the vial headspace and water together (54.7 + 1.2 + 16.8) to give a total of 72.7 nmol g^-1^. This conservative O_2_ concentration is between 10 and 50 × smaller than H_2_O_2_ concentrations after 1 week at 104°C in rock experiments (Figure 2, 3).

# 2. Supplementary Figures


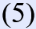

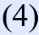

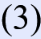

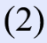

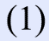

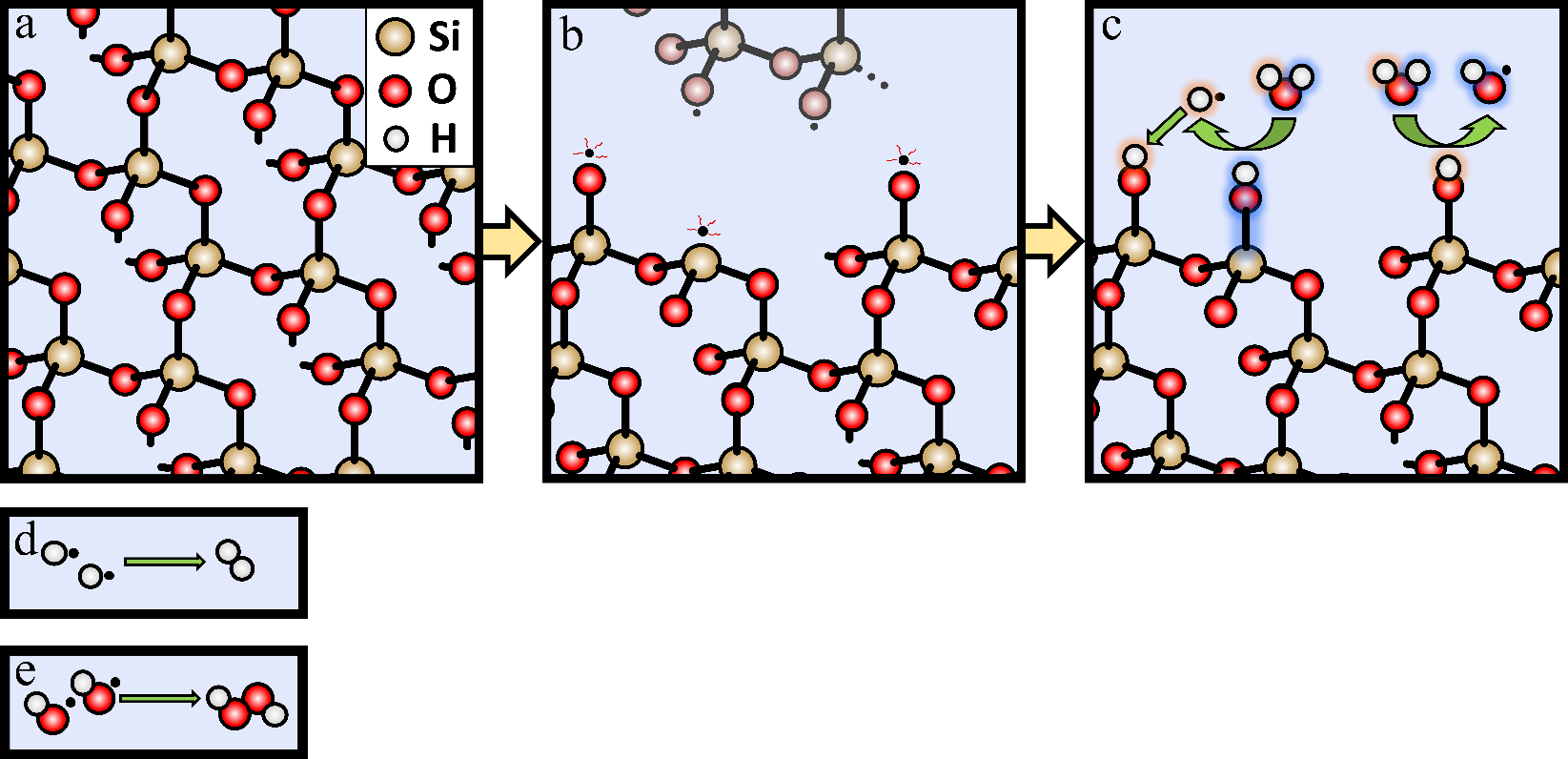


2≡Si• + 2H_2_O → 2≡SiOH + 2H• (1)

2H• → H_2_ (2)

≡SiO• + H• → ≡SiOH (3)

2≡SiO• + 2H_2_O → 2≡SiOH + 2•OH (4)

2•OH → H_2_O_2_ (5)

**Supplementary Figure 1**: Mechanochemical reactions relating to H_2_ and H_2_O_2_ production from cataclasis. (a) The structure of quartz. (b) Crushing rocks produces surface defects: Si• and SiO•. (c) Si• reacts with H_2_O to produce SiOH and H•. SiO• reacts with H• to produce SiOH, but also reacts with H_2_O to produce •OH and SiOH. (d) 2H• combine to form H_2_ (e) 2•OH combine to form H_2_O_2_.


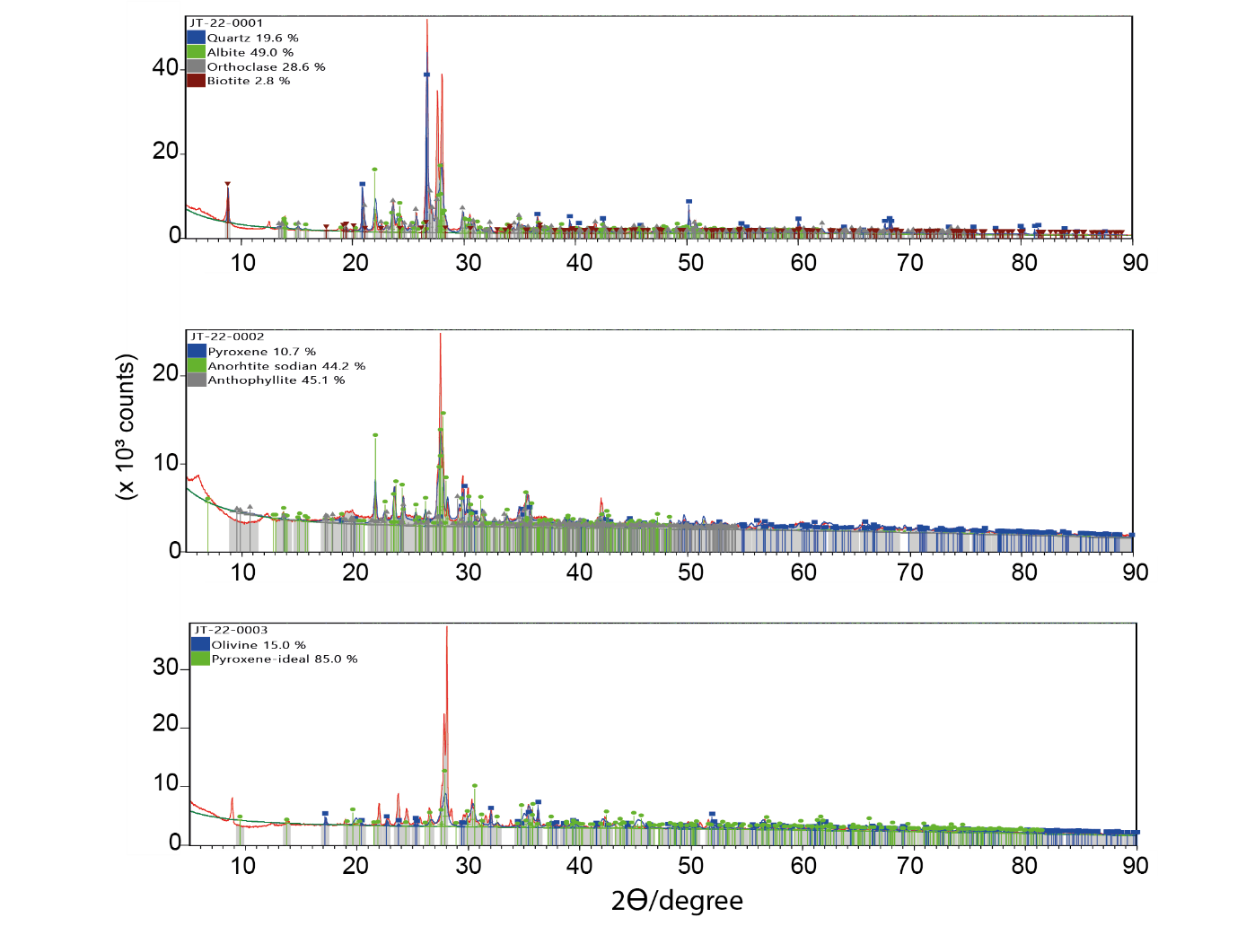


**Supplementary Figure 2**: Mineral composition of the three rock types used in the experiments. Figures show XRD scans and mineral identifications identified using the Rietveld method to identify major crystalline phases. Sample 1 (granite): quartz, albite, orthoclase, biotite. Sample 2 (basalt): pyroxene, anorthite, anthopyllite (amphibole). Sample 3 (peridotite): olivine, pyroxene.


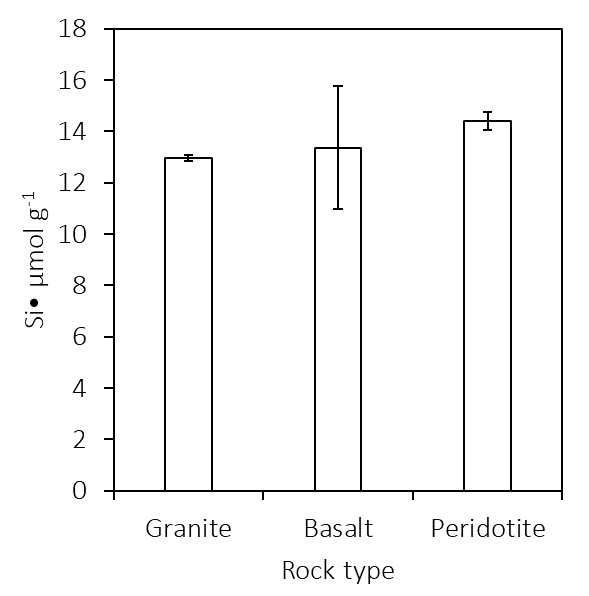


**Supplementary Figure 3:** The concentration of Si• on the surface of crushed granite, basalt, and peridotite crushed at 500 rpm for 30 min. The error bars are the standard error of the mean.


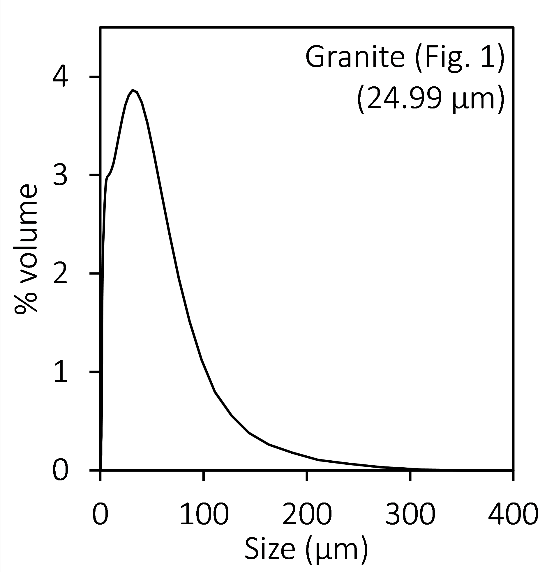

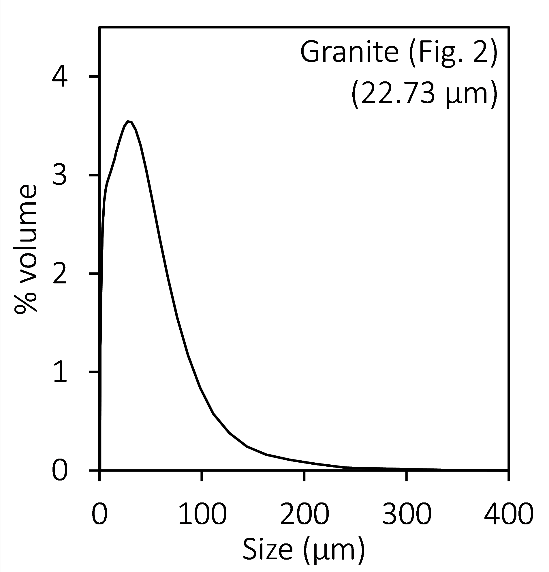


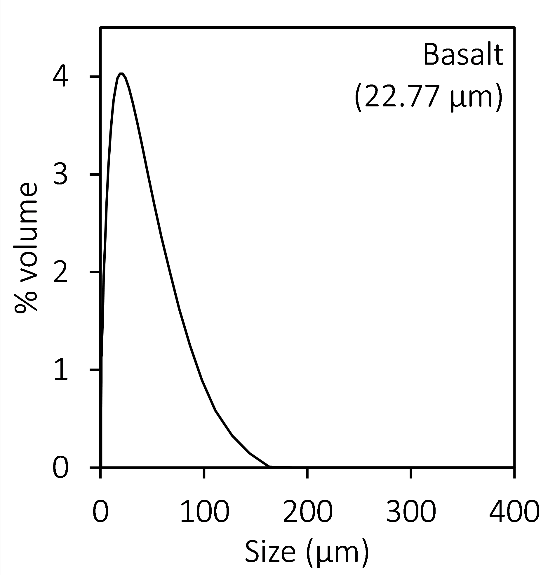

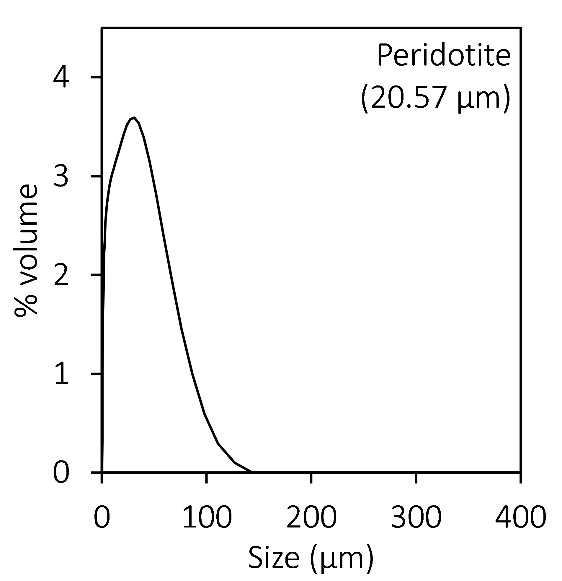


**Supplementary Figure** **4:** The grain size distributions and mean grain sizes (volume weighted) for all the rocks used in these experiments. The granite in the top left is the grain size for the data shown in Figure 1. All other rocks correspond to the rocks crushed for the data presented in Figures 2 & 3.


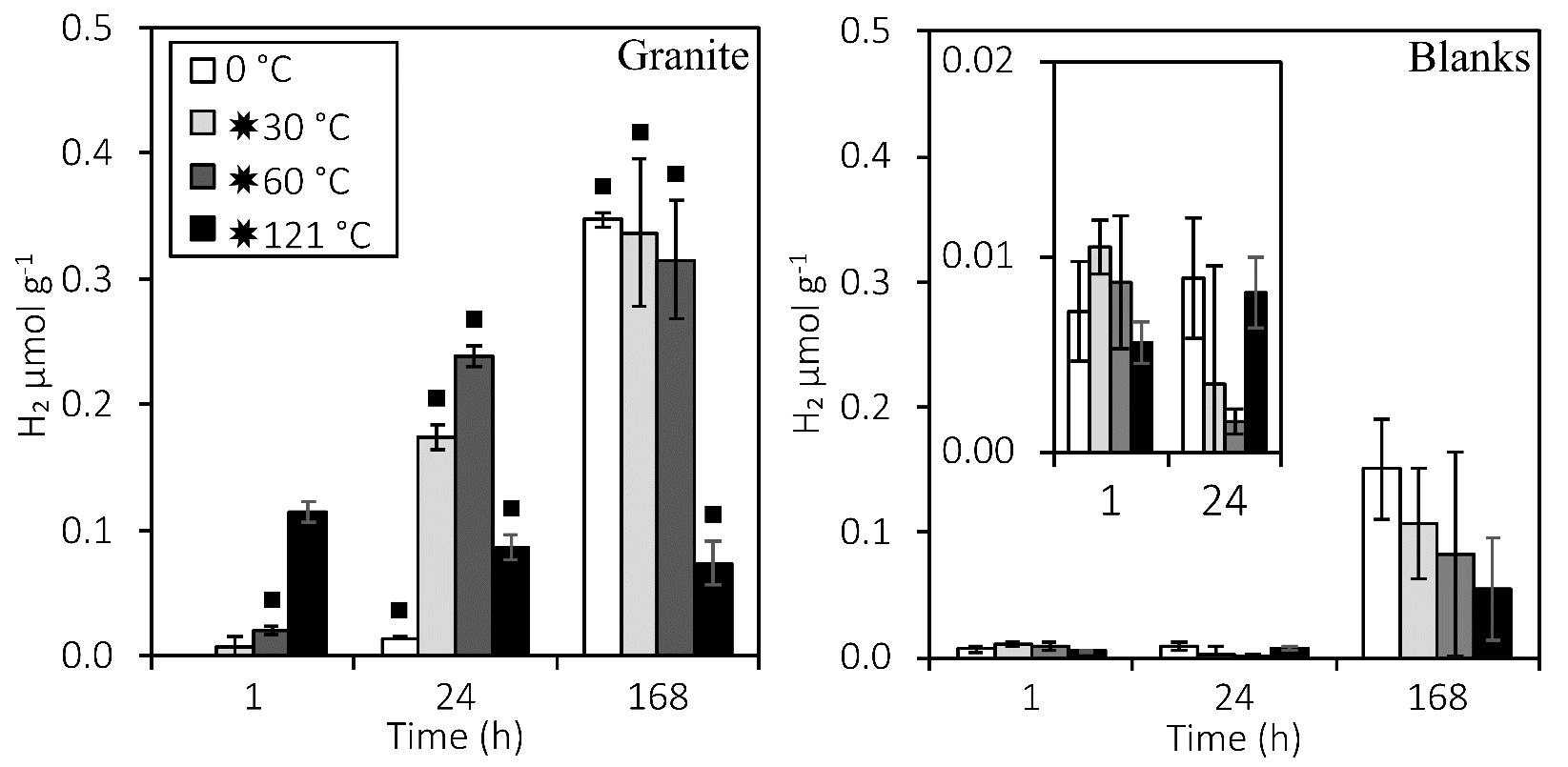


**Supplementary Figure 5:** H_2_ production from the blanks corresponding to Figure 1. The vials were filled with 4 mL anoxic water and flash heated to 30, 60, or 121 °C and incubated at 0 °C for either 1, 24, or 168 hrs. Time points are accurate to ±2 hrs. Error bars represent the standard error of the mean. The inset utilises the same axis titles.

**
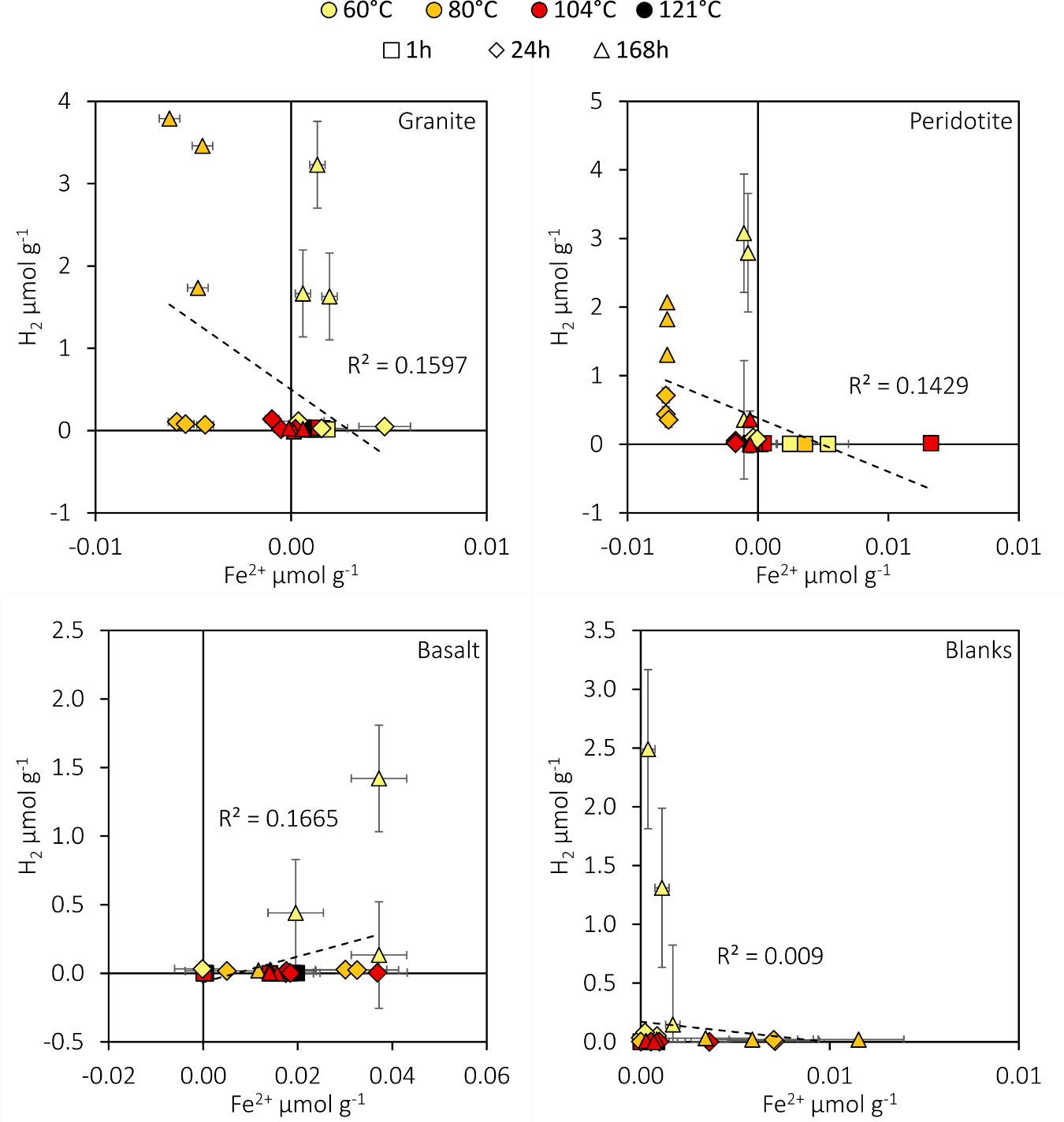
**

**Supplementary Figure 6:** Plots of Fe^2+^ and H_2_ produced in experiments with crushed basalt, peridotite and granite in water. All data are blank subtracted. Error bars are the standard deviation of the blanks multiplied by two. The experimental detection limits for Fe^2+^ and H_2_ are 0.004 and 0.00002 μmol g^-1^, respectively.


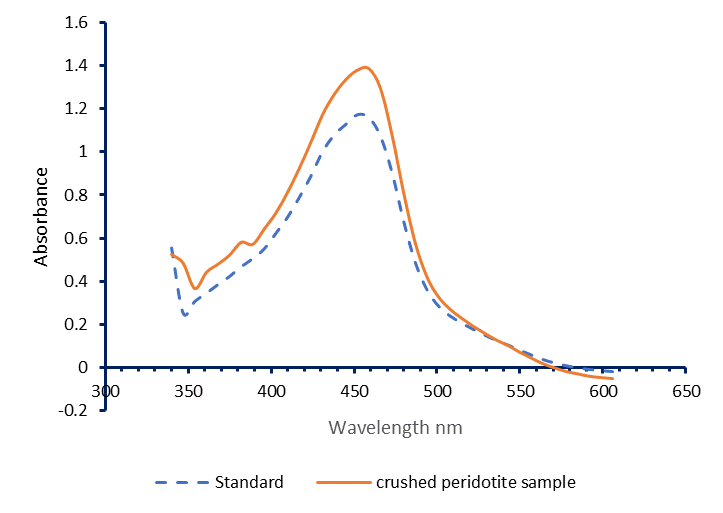


**Supplementary Figure 7**. Comparison of wavelength dependent adsorption spectra of a 200 μM H_2_O_2_ standard (blue dashed line) and aqueous extract of crushed peridotite crushed under N_2_ and incubated at 104°C for 1 week (red solid line).


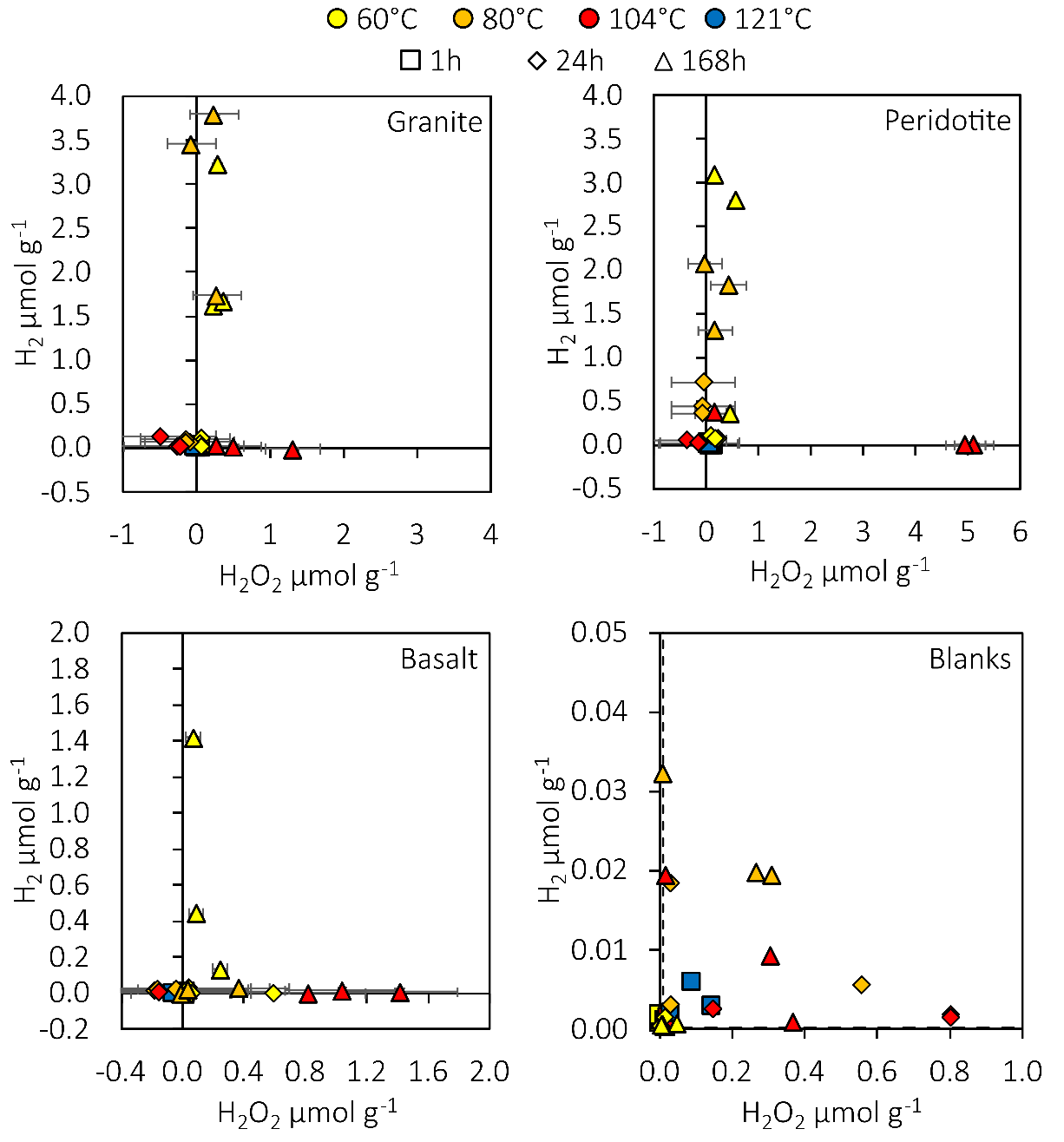


**Supplementary Figure 8:** Plots of H_2_ and H_2_O_2_ production by each rock type, including the blanks. The detection limits for H_2_ and H_2_O_2_ were to 0.0002 and 0.0092 μmol g^-1^, respectively. The 121v°C experiment was only measured after 1 hour, while the other temperatures show three time points: 1, 24, and 168 hrs. The error bars are 2× the standard deviation of the blanks at each temperature and time point. All data (except the blanks) is blank mean subtracted. The detection limits for H_2_ and H_2_O_2_ were 0.2 nmol g^-1^ and 9.2 nmol g^-1^, respectively.


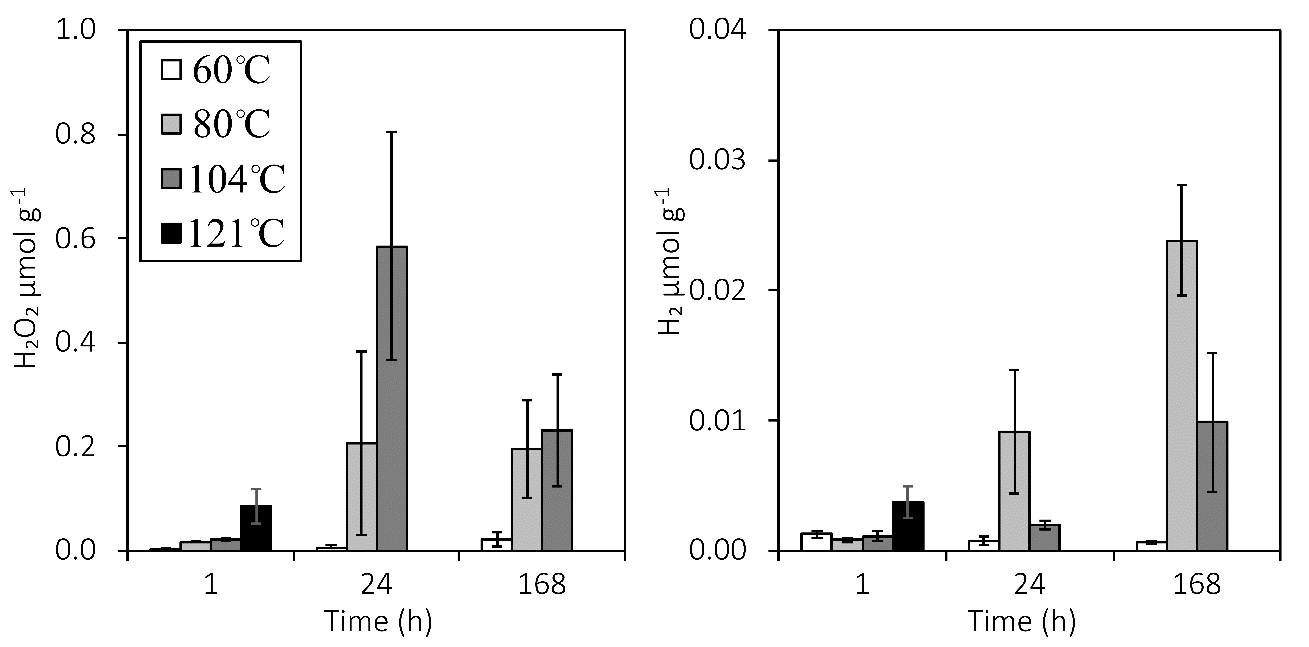


**Supplementary Figure** **9:** The H_2_O_2_ and H_2_ production from the blanks corresponding to Figure 2. Error bars are the standard error of the mean.


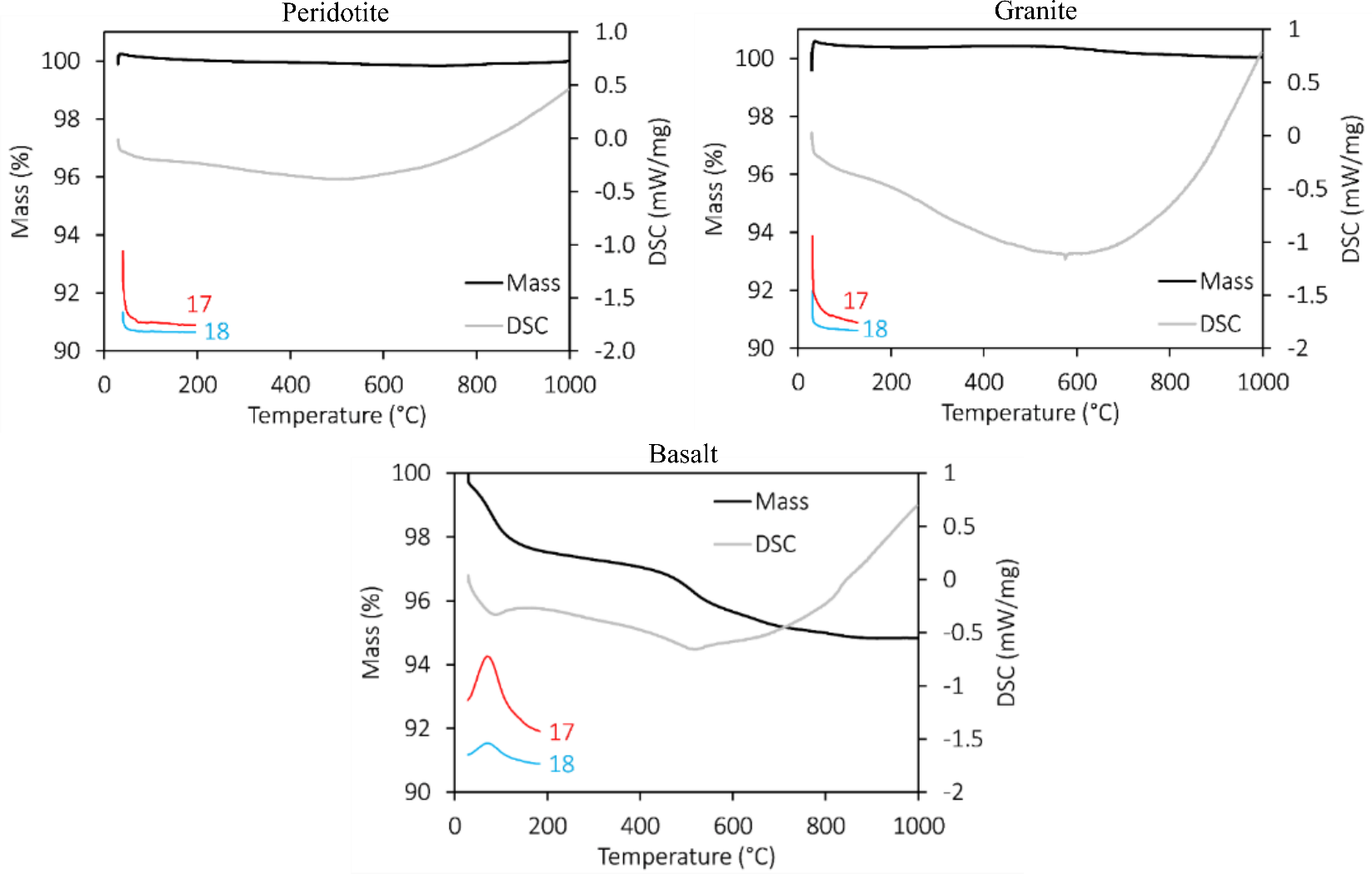


**Supplementary Figure** **10:** TGA results for basalt, granite, and peridotite showing a mass decrease at ~100 °C in basalt, coinciding with the detection of molecules with molecular weights of 17 and 18 (most likely OH and H_2_O within the structure of the rocks). DSC = Differential scanning calorimetry. The red and blue lines show the detection of molecules with molecular weights of 17 and 18, coinciding with a mass decrease of ~2%. The y axis for the DSC results (red and blue lines) is m/z (mass to charge ratio).


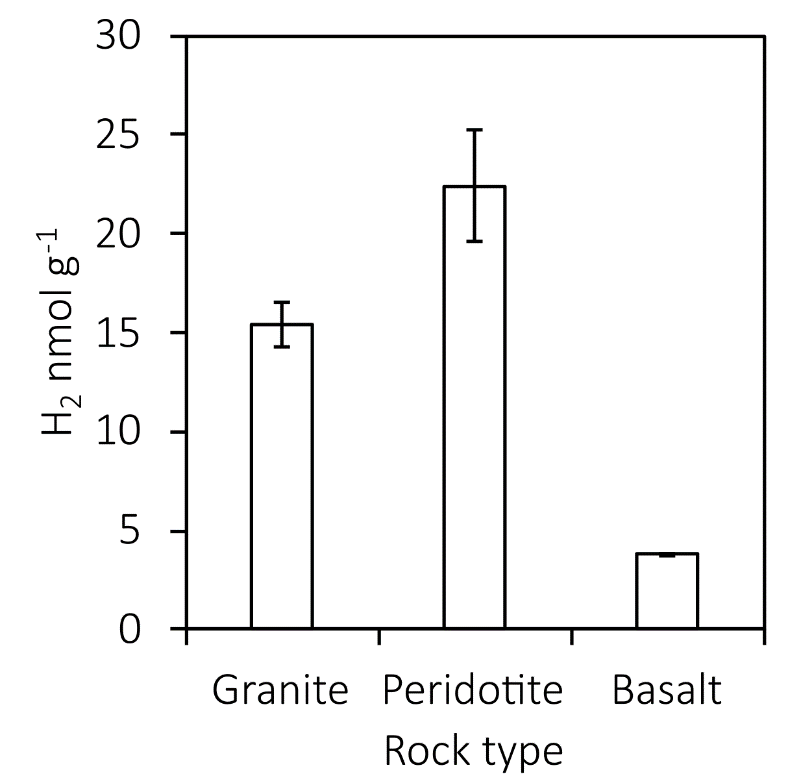


**Supplementary Figure 11:** Production of H_2_ from crushed rocks in the Planetary Ball Mill. Error bars are the standard error of the mean of three separate experiments where 45g of rock was crushed under N_~~2~~_ at 500 rpm for 30 min.


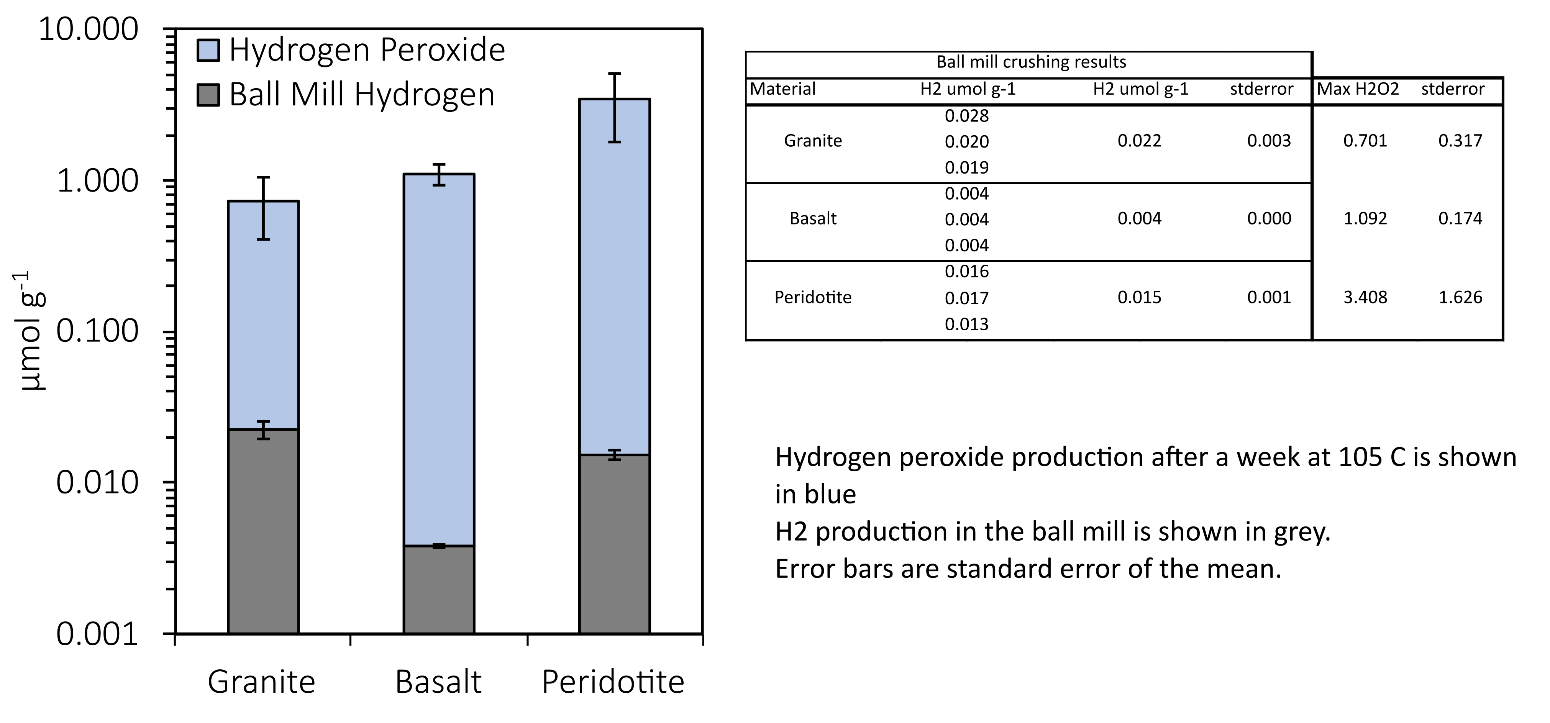


**Supplementary Figure 12:** Hydrogen production in the ball mill compared to the H_2_O_2_ production at 104 °C after a week – the ball mill H_2_ production (from the inferred reaction of Si•) cannot explain the SiO• surplus required for net H_2_O_2_ production. H_2_O_2_ production after a week at 104 °C is in blue. H_2_ production in the ball mill is in grey. Error bars are the standard error of the mean. Note the logarithmic scale.


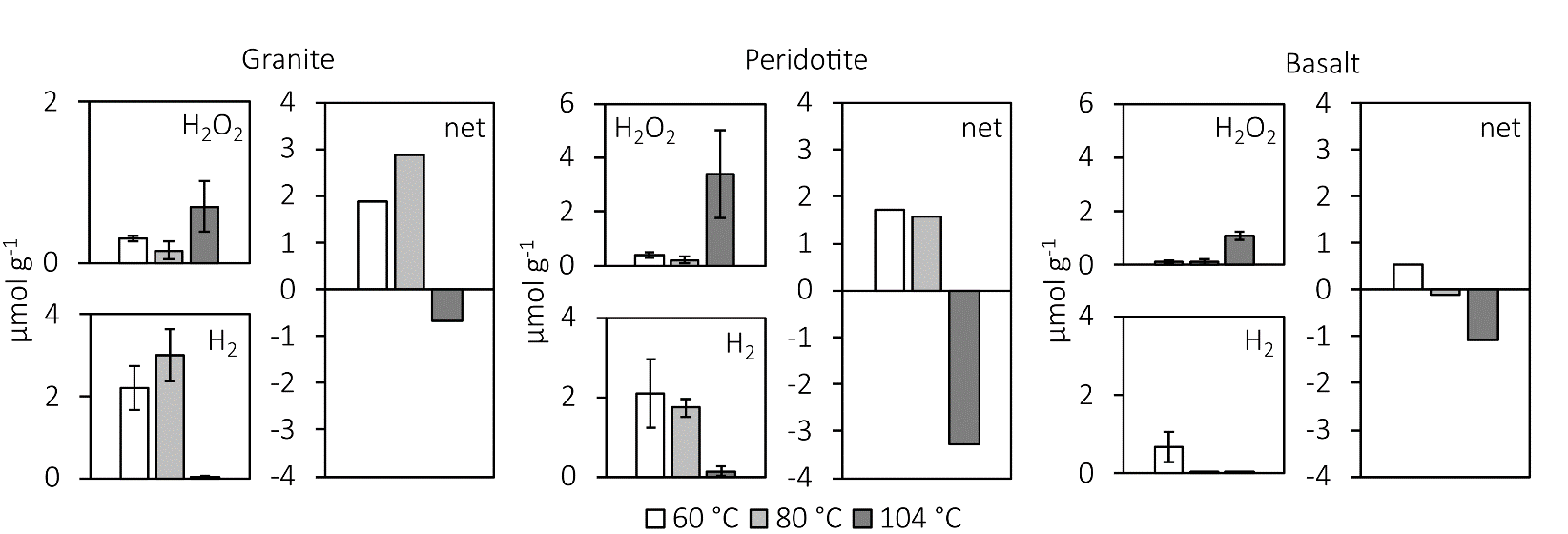


**Supplementary Figure 13:** The H_2_O_2_ and H_2_ produced in continuous heating experiments for crushed granite, peridotite and basalt after a week, and the net oxidant production. The net oxidant production is negative (i.e. net excess of oxidant) for all rock types at 104 °C. Error bars are the standard error of the mean. All vertical scales are μmol g^-1^.


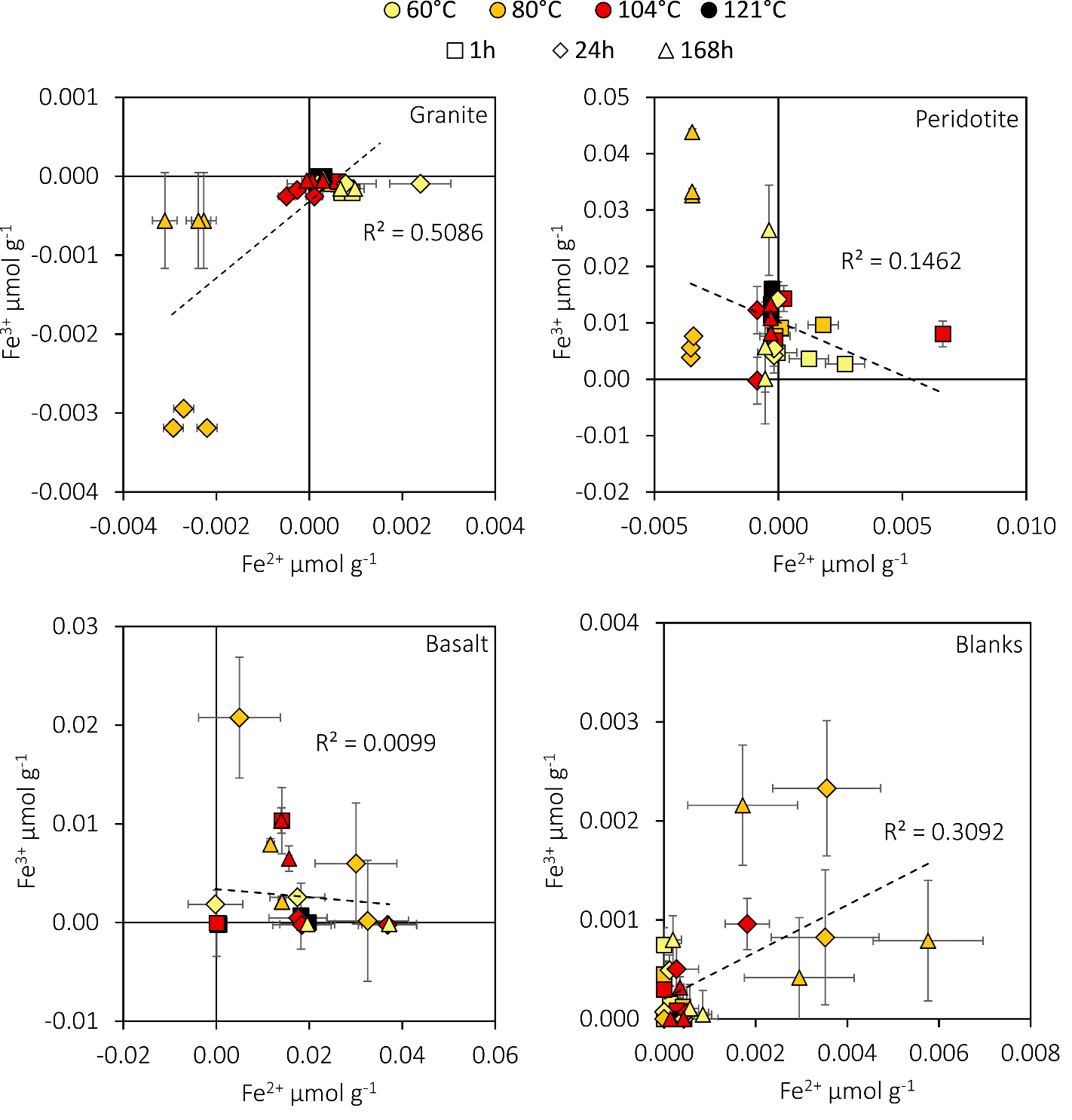


**Supplementary Figure 14:** Plots of Fe^2+^ and Fe^3+^ produced in experiments with crushed basalt, peridotite and granite in water. All data are blank subtracted. Error bars are the standard error of the mean. The experimental detection limits for Fe^2+^ and Fe^3+^ are 0.004 and 0.005 μmol g^-1^, respectively.


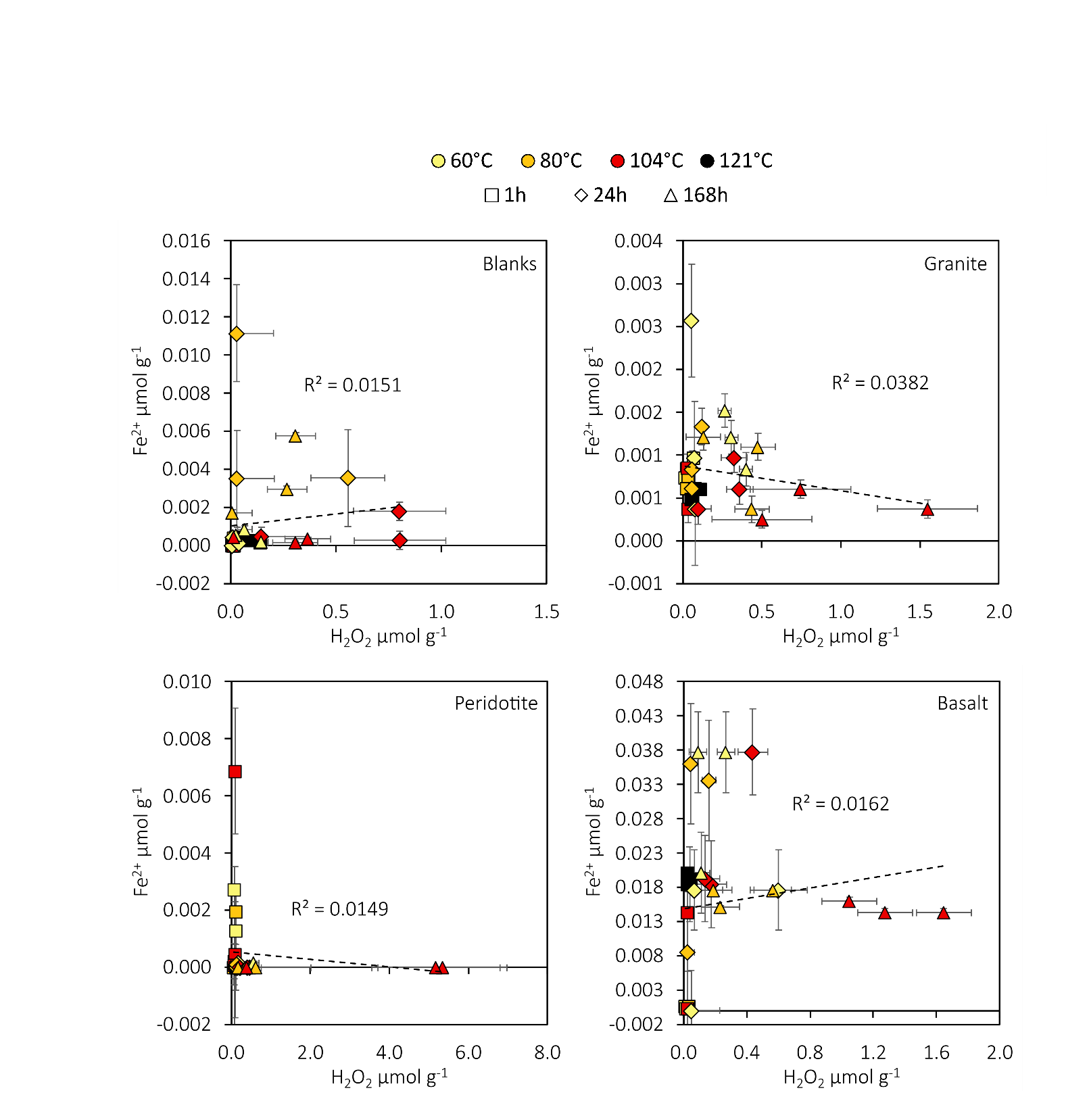


**Supplementary Figure 15**: Plots of Fe^2+^ and H_2_O_2_ produced in experiments with crushed basalt, peridotite and granite in water. All data are blank subtracted. Error bars are the standard deviation of the blanks multiplied by two. The experimental detection limits for Fe^2+^ and H_2_O_2_ are 0.004 and 9.2 nmol g^-1^ μmol g^-1^, respectively.


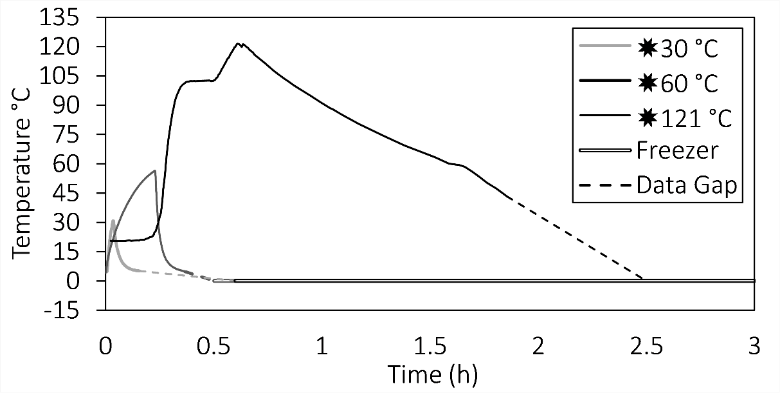


**Supplementary Figure 16**: A time series of the flash heating temperature changes. ✸ indicates flash heating. Temperature measurements were taken every two seconds for ✸30v°C and ✸60 °C and recorded manually for ✸121 °C every 30 sec.


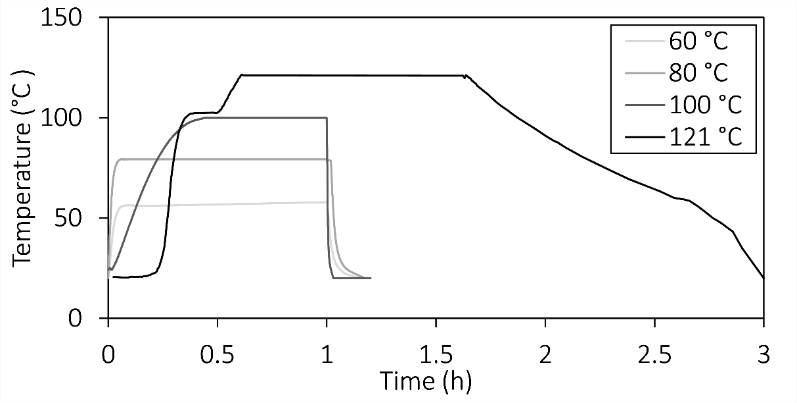


**Supplementary Figure 17**: Continuous heating temperature probe data for temperature change within the vials over the first hour. The data for 121 °C was not recorded for a 1 hr cycle of the autoclave, or for the decrease from ~60 °C down to 20 °C, so data from 80 °C and the flash heating experiment were used to fill in the gaps. For longer time points (24 and 168 hrs; excluding 121 °C), the time is prolonged at the respective temperature maximum. Variations in heating rate were caused by the use of different heating apparatus (water bath – 60 °C & 80 °C), oven (104 °C), and autoclave (121 °C).

# 3. Supplementary Tables

**Supplementary Table 2**: The mean concentrations and standard error of blanks. Detection limits (nmol g^-1^): H_2_O_2_ = 9.2, H_2_ = 0.2, •OH = 29.46, Fe^2+^ = 0.004, Fe^3+^ = 0.005.

| Blanks Mean | | | | | | | | |
| --- | --- | --- | --- | --- | --- | --- | --- | --- |
| Experiment | Time (h) | Temp (°C) | H_2_O_2_ | H_2_ | pH | •OH | Fe^2+^ | Fe^3+^ |
|  |  |  | μmol g^-1^ | μmol g^-1^ |  | μmol g^-1^ | μmol g^-1^ | μmol g^-1^ |
| Blanks (Fig. 2) | 1 | 60 | 0.003 | 0.001 | 7.7 | 0.008 | <0.0001 | 0.0004 |
|  | 1 | 80 | 0.018 | 0.001 | 8.7 | 0.017 | 0.0001 | 0.0002 |
|  | 1 | 104 | 0.022 | 0.001 | 8.0 | 0.008 | 0.0002 | 0.0001 |
|  | 3 | 121 | 0.086 | 0.004 | 9.0 | 0.009 | 0.0003 | <0.0001 |
|  | 24 | 60 | 0.007 | 0.001 | 8.0 | 0.009 | 0.0002 | 0.0002 |
|  | 24 | 80 | 0.206 | 0.009 | 9.0 | 0.008 | 0.0061 | 0.0064 |
|  | 24 | 104 | 0.584 | 0.002 | 10.0 | 0.016 | 0.0009 | 0.0005 |
|  | 167 | 61 | 0.022 | 0.001 | 7.2 | 0.010 | 0.0005 | 0.0003 |
|  | 168 | 80 | 0.195 | 0.024 | 8.8 | 0.009 | 0.0035 | 0.0011 |
|  | 168 | 104 | 0.231 | 0.010 | 9.0 | 0.008 | 0.0003 | 0.0001 |
| Blanks (Fig. 1) | 1 | 0 |  | 0.004 |  |  |  |  |
|  | 1 | 30 |  | 0.006 |  |  |  |  |
|  | 1 | 60 |  | 0.005 |  |  |  |  |
|  | 1 | 121 |  | 0.003 |  |  |  |  |
|  | 24 | 0 |  | 0.005 |  |  |  |  |
|  | 24 | 30 |  | 0.009 |  |  |  |  |
|  | 24 | 60 |  | 0.001 |  |  |  |  |
|  | 24 | 121 |  | 0.005 |  |  |  |  |
|  | 168 | 0 |  | 0.088 |  |  |  |  |
|  | 168 | 30 |  | 0.063 |  |  |  |  |
|  | 168 | 60 |  | 0.049 |  |  |  |  |
|  | 168 | 121 |  | 0.032 |  |  |  |  |
| Blanks Standard Error of the mean | | | | | | | | |
| Blanks (Fig. 2) | 1 | 60 | 0.002 | <0.001 | <0.001 | <0.001 | <0.0001 | 0.0002 |
|  | 1 | 80 | 0.002 | <0.001 | <0.001 | 0.009 | 0.0001 | 0.0001 |
|  | 1 | 104 | 0.003 | <0.001 | <0.001 | 0.001 | 0.0001 | 0.0001 |
|  | 3 | 121 | 0.033 | 0.001 | <0.001 | 0.001 | 0.0001 | <0.0001 |
|  | 24 | 60 | 0.004 | <0.001 | <0.001 | <0.001 | 0.0001 | 0.0002 |
|  | 24 | 80 | 0.176 | 0.005 | 0.005 | <0.001 | 0.0025 | 0.0048 |
|  | 24 | 104 | 0.219 | <0.001 | <0.001 | 0.007 | 0.0005 | 0.0003 |
|  | 167 | 61 | 0.014 | <0.001 | <0.001 | 0.001 | 0.0002 | 0.0002 |
|  | 168 | 80 | 0.094 | 0.004 | 0.001 | <0.001 | 0.0012 | 0.0005 |
|  | 168 | 104 | 0.108 | 0.005 | <0.001 | 0.001 | 0.0001 | 0.0001 |
| Blanks (Fig. 1) | 1 | 0 |  | 0.001 |  |  |  |  |
|  | 1 | 30 |  | 0.001 |  |  |  |  |
|  | 1 | 60 |  | 0.002 |  |  |  |  |
|  | 1 | 121 |  | 0.001 |  |  |  |  |
|  | 24 | 0 |  | 0.002 |  |  |  |  |
|  | 24 | 30 |  | 0.004 |  |  |  |  |
|  | 24 | 60 |  | 0.000 |  |  |  |  |
|  | 24 | 121 |  | 0.001 |  |  |  |  |
|  | 168 | 0 |  | 0.023 |  |  |  |  |
|  | 168 | 30 |  | 0.026 |  |  |  |  |
|  | 168 | 60 |  | 0.048 |  |  |  |  |
|  | 168 | 121 |  | 0.024 |  |  |  |  |

**Supplementary Table 3**: The blank subtracted mean concentrations of the chemistry measured from crushed rocks experiments. All concentrations are reported in μmol g^-1^. Detection limits (nmol g^-1^): H_2_O_2_ = 9.2, H_2_ = 0.2, •OH = 29.46, Fe^2+^ = 0.004, Fe^3+^ = 0.005.

| All Blank subtracted means | | | | | | |  |  |
| --- | --- | --- | --- | --- | --- | --- | --- | --- |
| Experiment | Time (h) | Temp (°C) | H_2_O_2_ | H_2_ | pH | •OH | Fe^2+^ | Fe^3+^ |
|  |  |  | μmol g^-1^ | μmol g^-1^ |  | μmol g^-1^ | μmol g^-1^ | μmol g^-1^ |
| Granite (Fig. 2) | 1 | 60 | 0.035 | 0.022 | 10.0 | 0.001 | 0.001 | <0.001 |
|  | 1 | 80 | 0.029 | 0.016 | 8.5 | -0.011 | <0.001 | <0.001 |
|  | 1 | 104 | 0.010 | 0.032 | 8.0 | -0.001 | <0.001 | <0.001 |
|  | 3 | 121 | -0.008 | 0.031 | 9.0 | -0.004 | <0.001 | <0.001 |
|  | 24 | 60 | 0.063 | 0.063 | 8.2 | 0.004 | 0.001 | <0.001 |
|  | 24 | 80 | -0.125 | 0.085 | 8.3 | 0.001 | 0.001 | <0.001 |
|  | 24 | 104 | -0.323 | 0.059 | 8.3 | -0.016 | 0.001 | <0.001 |
|  | 167 | 61 | 0.303 | 2.175 | 9.0 | -0.001 | 0.001 | <0.001 |
|  | 168 | 80 | 0.152 | 2.995 | 8.0 | 0.002 | 0.001 | <0.001 |
|  | 168 | 104 | 0.701 | 0.011 | 8.3 | <0.001 | <0.001 | <0.001 |
| Peridotite (Fig. 2) | 1 | 61 | 0.108 | 0.007 | 9.7 | 0.006 | 0.001 | 0.004 |
|  | 1 | 80 | 0.071 | 0.007 | 9.5 | -0.006 | 0.001 | 0.009 |
|  | 1 | 104 | 0.073 | 0.015 | 9.0 | -0.001 | 0.002 | 0.010 |
|  | 3 | 121 | 0.047 | 0.014 | 9.0 | -0.002 | <0.001 | 0.014 |
|  | 24 | 61 | 0.167 | 0.083 | 9.5 | 0.001 | <0.001 | 0.008 |
|  | 24 | 80 | -0.062 | 0.503 | 9.0 | 0.002 | <0.001 | 0.009 |
|  | 25 | 104 | -0.221 | 0.030 | 9.0 | -0.004 | <0.001 | 0.004 |
|  | 167 | 61 | 0.383 | 2.076 | 9.3 | -0.005 | <0.001 | 0.011 |
|  | 167 | 80 | 0.187 | 1.734 | 9.0 | <0.001 | <0.001 | 0.037 |
|  | 167 | 104 | 3.408 | 0.119 | 9.0 | -0.001 | <0.001 | 0.011 |
| Basalt (Fig. 2) | 1 | 61 | 0.013 | 0.003 | 7.8 | 0.001 | <0.001 | <0.001 |
|  | 1 | 80 | 0.014 | 0.002 | 7.7 | -0.010 | <0.001 | <0.001 |
|  | 1 | 105 | 0.009 | 0.002 | 8.2 | -0.002 | <0.001 | <0.001 |
|  | 3 | 121 | -0.053 | 0.003 | 8.0 | -0.003 | <0.001 | <0.001 |
|  | 24 | 61 | 0.234 | 0.013 | 7.5 | 0.002 | 0.012 | 0.002 |
|  | 24 | 80 | -0.129 | 0.023 | 7.3 | -0.002 | 0.026 | 0.012 |
|  | 24 | 105 | -0.335 | 0.008 | 7.7 | -0.010 | 0.025 | <0.001 |
|  | 168 | 61 | 0.135 | 0.665 | 7.5 | -0.002 | 0.032 | <0.001 |
|  | 168 | 80 | 0.134 | 0.016 | 7.5 | -0.002 | 0.017 | 0.005 |
|  | 168 | 105 | 1.092 | 0.006 | 8.0 | 0.001 | 0.015 | 0.009 |
| Granite (Fig. 1) | 1 | 0 |  | -0.002 |  |  |  |  |
|  | 1 | 30 |  | 0.006 |  |  |  |  |
|  | 1 | 60 |  | 0.020 |  |  |  |  |
|  | 1 | 121 |  | 0.115 |  |  |  |  |
|  | 24 | 0 |  | 0.015 |  |  |  |  |
|  | 24 | 30 |  | 0.174 |  |  |  |  |
|  | 24 | 60 |  | 0.238 |  |  |  |  |
|  | 24 | 121 |  | 0.087 |  |  |  |  |
|  | 168 | 0 |  | 0.345 |  |  |  |  |
|  | 168 | 30 |  | 0.335 |  |  |  |  |
|  | 168 | 60 |  | 0.314 |  |  |  |  |
|  | 168 | 121 |  | 0.073 |  |  |  |  |

**Supplementary Table 4**: The standard error of the mean of the chemistry measured from crushed rock experiments corresponding to the means presented in Supplementary Table 3.

| All Blank subtracted standard error | | | | | | | | |
| --- | --- | --- | --- | --- | --- | --- | --- | --- |
| Experiment | Time (h) | Temp (°C) | H_2_O_2_ | H_2_ | pH | •OH | Fe^2+^ | Fe^3+^ |
|  |  |  | μmol g^-1^ | μmol g^-1^ |  | μmol g^-1^ | μmol g^-1^ | μmol g^-1^ |
| Granite (Fig. 2) | 1 | 60 | 0.017 | 0.004 | 0.000 | 0.000 | 0.0001 | 0.0000 |
|  | 1 | 80 | 0.016 | 0.003 | 0.408 | 0.002 | 0.0000 | 0.0000 |
|  | 1 | 104 | 0.002 | 0.003 | 0.000 | 0.000 | 0.0002 | 0.0000 |
|  | 3 | 121 | 0.017 | 0.003 | 0.000 | 0.002 | 0.0000 | 0.0000 |
|  | 24 | 60 | 0.007 | 0.026 | 0.441 | 0.001 | 0.0007 | 0.0000 |
|  | 24 | 80 | 0.021 | 0.011 | 0.333 | 0.001 | 0.0002 | 0.0001 |
|  | 24 | 104 | 0.082 | 0.040 | 0.333 |  | 0.0002 | 0.0000 |
|  | 167 | 61 | 0.040 | 0.527 | 0.577 | 0.001 | 0.0002 | 0.0000 |
|  | 168 | 80 | 0.109 | 0.637 | 0.577 | 0.001 | 0.0003 | 0.0000 |
|  | 168 | 104 | 0.317 | 0.010 | 0.333 | 0.002 | 0.0001 | 0.0000 |
| Peridotite (Fig. 2) | 1 | 61 | 0.014 | 0.002 | 0.333 |  | 0.0008 | 0.0006 |
|  | 1 | 80 | 0.016 | 0.000 | 0.289 | 0.001 | 0.0006 | 0.0006 |
|  | 1 | 104 | 0.005 | 0.002 | 0.000 | 0.001 | 0.0022 | 0.0023 |
|  | 3 | 121 | 0.010 | 0.001 | 0.289 | 0.002 | 0.0000 | 0.0014 |
|  | 24 | 61 | 0.039 | 0.007 | 0.408 | 0.001 | 0.0001 | 0.0031 |
|  | 24 | 80 | 0.006 | 0.108 | 0.000 | 0.001 | 0.0000 | 0.0011 |
|  | 25 | 104 | 0.078 | 0.011 | 0.000 | 0.001 | 0.0000 | 0.0042 |
|  | 167 | 61 | 0.118 | 0.863 | 0.167 | 0.003 | 0.0001 | 0.0080 |
|  | 167 | 80 | 0.131 | 0.226 | 0.289 | 0.002 | 0.0000 | 0.0036 |
|  | 167 | 104 | 1.626 | 0.122 | 0.289 | 0.001 | 0.0000 | 0.0015 |
| Basalt (Fig. 2) | 1 | 61 | 0.002 | 0.001 | 0.167 | 0.001 | 0.0001 | 0.0000 |
|  | 1 | 80 | 0.002 | 0.001 | 0.167 | 0.001 | 0.0001 | 0.0000 |
|  | 1 | 105 | 0.004 | 0.000 | 0.441 | 0.003 | 0.0055 | 0.0034 |
|  | 3 | 121 | 0.009 | 0.000 | 0.000 | 0.001 | 0.0005 | 0.0002 |
|  | 24 | 61 | 0.181 | 0.010 | 0.289 | 0.003 | 0.0059 | 0.0002 |
|  | 24 | 80 | 0.042 | 0.003 | 0.333 | 0.001 | 0.0088 | 0.0061 |
|  | 24 | 105 | 0.094 | 0.005 | 0.167 | 0.000 | 0.0063 | 0.0002 |
|  | 168 | 61 | 0.056 | 0.388 | 0.289 | 0.001 | 0.0059 | 0.0000 |
|  | 168 | 80 | 0.119 | 0.008 | 0.289 | 0.001 | 0.0008 | 0.0019 |
|  | 168 | 105 | 0.174 | 0.005 | 0.000 | 0.002 | 0.0006 | 0.0013 |
| Granite (Fig. 1) | 1 | 0 |  | 0.001 |  |  |  |  |
|  | 1 | 30 |  | 0.010 |  |  |  |  |
|  | 1 | 60 |  | 0.005 |  |  |  |  |
|  | 1 | 121 |  | 0.007 |  |  |  |  |
|  | 24 | 0 |  | 0.002 |  |  |  |  |
|  | 24 | 30 |  | 0.009 |  |  |  |  |
|  | 24 | 60 |  | 0.009 |  |  |  |  |
|  | 24 | 121 |  | 0.009 |  |  |  |  |
|  | 168 | 0 |  | 0.028 |  |  |  |  |
|  | 168 | 30 |  | 0.069 |  |  |  |  |
|  | 168 | 60 |  | 0.094 |  |  |  |  |
|  | 168 | 121 |  | 0.033 |  |  |  |  |

**Supplementary Table 5**: Mass changes in agate ball mill (including lid and grinding balls) after milling for 30 min at 500 rpm in a Fritsch P6 planetary ball mill. Precision of weighing scales was ± 0.1 g. n = 1 for each material.

| Milling material | Mass before milling (g) | Mass after milling (g) | Difference in mass (g) | % difference |
| --- | --- | --- | --- | --- |
| 45 g Granite | 1784.0 | 1784.0 | 0.0 | 0.0 |
| 45 g Peridoitite | 1784.0 | 1783.9 | -0.1 | -0.2 |
| 45 g Basalt | 1783.9 | 1784.0 | +0.1 | +0.2 |
